# Resistance of *Enterococcus* spp. in Dust From Farm Animal Houses: A Retrospective Study

**DOI:** 10.3389/fmicb.2018.03074

**Published:** 2018-12-13

**Authors:** Mengda Liu, Nicole Kemper, Nina Volkmann, Jochen Schulz

**Affiliations:** Institute for Animal Hygiene, Animal Welfare and Farm Animal Behavior, University of Veterinary Medicine Hannover, Foundation, Hannover, Germany

**Keywords:** *Enterococcus*, survival, dust, livestock, resistance

## Abstract

In a retrospective study, the antimicrobial susceptibility of *Enterococcus* spp. isolated from stored sedimentation dust samples from cattle, pig and poultry barns to 16 antibiotics was determined using a microdilution test. The resistance phenotypes of 70 isolates from different timespans (8 from the 1980s, 15 from the 1990s, 43 from the 2000s and 4 from 2015) were determined. Resistant enterococci were detected in samples from all time periods. Resistances to three or more antibiotics occurred in 69 percent of all isolates. The oldest multidrug resistant isolate was an *Enterococcus faecium* obtained from a 35-year-old pig barn dust sample. No correlations (ρ = 0.16, *p* = 0.187) were found between the age of isolates and the number of resistances. Instead, the number of resistances was associated with the origin of the isolates. An exact logistic conditional regression analysis showed significant differences in resistance to ciprofloxacin, erythromycin, penicillin and tylosin between isolates from different animal groups. Interestingly, we isolated ciprofloxacin-resistant *E. faecium* from pig barn dust before fluoroquinolones were introduced into the market for use in animal husbandry. In conclusion, dust from farm animal houses is a reservoir and carrier of multidrug-resistant *Enterococcus* spp. People working in barns are unavoidably exposed to these bacteria. Furthermore, it can be hypothesized that emissions from barns of intensive livestock farming contaminate the environment with multidrug resistant enterococci.

## Introduction

*Enterococcus* spp. can be found in the gut microbiota of mammals and birds and are opportunistic pathogens (Byappanahalli et al., 2012). *Enterococcus* spp. can infect farm animals and cause nosocomial infections in humans (Byappanahalli et al., 2012). Although *Enterococcus* spp. are predominately adapted to their hosts, transmission between animals and humans has been described and is a risk factor for the spread of these organisms (Lu et al., 2002; Kataoka et al., 2014; Lebreton et al., 2014; Milton et al., 2015). Furthermore, the horizontal transfer of resistance genes from animal strains to pathogenic human strains is considered a human hazard (Hammerum, 2012).

One pool of transmissible strains and resistance genes seems to be farm animals (Lu et al., 2002; Donabedian et al., 2006). For instance, in a comprehensive study, Hershberger et al. (2005) showed that farm animals were a reservoir of antibiotic-resistant enterococci and that resistance was more common on farms using antimicrobials. Such strains from animals are potentially able to transfer resistance genes to pathogenic bacteria. This concern is one of the reasons why trends of resistances in animal isolates are monitored in member states of the European Union and other areas (EFSA, 2015).

Isolates for monitoring programs are commonly obtained from animals, meat, and fecal samples. Furthermore, enterococci are suggested as useful indicator organisms for fecal contaminations of the environment because of their relatively high tenacity outside their hosts (Lukasova and Sustackova, 2003). Since fecal particles are a component of dust in animal housing they could be a source of fecal bacteria (Schulz et al., 2016). This fact explains, for instance, the detection of vancomycin-resistant enterococci (VRE) in dust samples from turkey flocks (Sting et al., 2013). Evidence also suggests that dust from farm animal houses might be a reservoir for multidrug-resistant fecal enterococci, as shown for fecal *Enterobacteriaceae* (Schulz et al., 2016).

Therefore, this retrospective study analyzed the occurrence of enterococci in 125 dust samples from cattle, pig, and poultry barns and the resistance profiles of these bacteria. The dust samples originated from different investigations and studies conducted between 1980 and 2015. During this time span, fluoroquinolones were introduced in the market for use in animal husbandry (Guardabassi et al., 2008). In the same time span, the use of antibiotics as growth promoters was forbidden (Wegener et al., 1999). These events might have had an impact on the resistances of isolates from animal husbandry. Antimicrobial susceptibility testing to determine resistance profiles could be a useful tool in assessing the impacts on the antimicrobial resistances of isolates from farm animal houses (Wiedemann and Heisig, 1999).

## Materials and Methods

### Origins of Dust Samples

From 1980 to 2009, 125 dust samples were collected by sedimentation in Northern Germany. The samples originate from five pig houses, eight poultry barns, and one cattle barn. The samples were taken as parts of various studies. The sedimentation dust samples were collected and stored as described by Schulz et al. (2016). Briefly, collected sedimentation dust samples (between 5 and 50 g) were stored in sterile glass cylinders subsequently sealed with sterile corks and stored in an air-conditioned room at 4°C in the dark.

Additionally, five pooled dust samples collected from a broiler barn in 2015 were included in the study. Dust was transferred by sterile brushes into sterile bags from different dusty surfaces in a barn. After transport to the laboratory, the dust samples were also transferred in sterile glass cylinders. However, a freezer was used to store the samples at 4°C in the dark.

### Isolation and Identification of *Enterococcus* spp.

Dust suspensions were prepared as described by Schulz et al. (2016). Subsequently, aliquots (0.1 ml and 0.1 ml of a tenfold dilution and 0.1 ml of a hundred-fold dilution) were plated in triplicate on Bile Aesculin Agar (BAA) (Oxoid Deutschland GmbH, Wesel, Germany) and on BAA supplemented with ciprofloxacin at 4 mg/L (BAACIP) (CIP: Sigma-Aldrich Chemie GmbH, Steinheim, Germany). The detection limit was 1,000 cfu/g of dust. The buffer used to prepare the dust suspensions was plated as a negative control. *Enterococcus faecium* (DSM 2918) and *Enterococcus fecalis* (DSM 20478) were streaked on BAA as growth controls. The plates were incubated at 37°C for 48 h. Presumed enterococci colonies appear with diameters of 1–2 mm and are usually larger than common streptococci, shiny in appearance, and brown with brown or black halos on BAA (Public Health England, 2014; Thermofisher.com, 2017).

At least two putative enterococci colonies of every cultivable sample were randomly selected, streaked on Columbia Agar with sheep blood (COLSB) (Oxoid Deutschland GmbH, Wesel, Germany), and identified as described in the thesis from Liu (2017). Briefly, presumed *Enterococcus* spp. isolates were incubated on API® 20 STREP biochemical test strips in accordance with the manufacturer's protocol (bioMérieux SA, Marcy-l'Étoile, France). After 24 h of incubation, results were analyzed using the apiweb™–API 20 STREP V7.0 software (bioMérieux, Deutschland GmbH, Germany). When the probability of identification was more than 90%, the result was seen as confirmed. However, the differentiation of species from the *E. faecium* group by biochemical tests can fail (Devrise et al., 2002). Therefore, a molecular biological method was used to identify isolates to species level (Stepień-Pyśniak et al., 2017). Stored isolates (at minus 80°C) were analyzed by matrix-assisted laser desorption/ionization time of flight mass spectrometry (MALDI-TOF MS). Isolates were incubated on CLOSB at 37°C overnight, afterwards being analyzed by Bruker MALDI Biotyper (Bruker Daltonics, Billerica, USA) in accordance with the manufacturer's protocol. Identified species are summarized in Table 1. More detailed results (log(score) values) are shown in the Supplementary Table 1.

**Table 1:** Origin and number of isolated species.

**Year of sampling**	**Number of isolates (origin)**	
	***Enterococcus faecium*** **(*****n*** **=** **64)**		***Other enterococcus* spp. (*n* = 6)**
	**From BAA**	**From BAACIP**	**From BAA**
1981	1 (pig barn)	
1984	2 (pig barn)	
1988	4 (pig barn)	
1989			1 *E. hirae* (pig barn)
1992	1 (pig barn)	
1993	1 (pig barn)	
1994	1 (broiler barn)	6 (broiler barn)	1 *E. hirae* (pig barn)
1995	1 (pig barn)	
1996	1 (pig barn)	
1997			1 *E. hirae* (pig barn)
1998			1 *E. hirae* (pig barn)
1999			1 *E. hirae* (pig barn)
2003	1 (duck barn)	
2004	3 (broiler barn); 1 (turkey barn)	2 (turkey barn)
2005	2 (pig barn); 3 (cattle barn)	6 (pig barn); 10 (broiler barn); 9 (laying hen house)
2009	5 (pig barn)	1 (pig barn)
2015	3 (broiler barn)		1 *E. casseliflavus* (broiler barn)

### Antimicrobial Susceptibility

An antimicrobial sensitivity test was performed by the microdilution method for all confirmed enterococci isolates. The code of the commercially prepared microdilution panels is CMV3AGPF (Thermo Fisher Scientific Inc., Waltham, USA). The 15 antibiotics tested were tigecycline (TGC), tetracycline (TET), chloramphenicol (CHL), daptomycin (DAP), streptomycin (STR), tylosin (TYLT), quinupristin/dalfopristin (synercid) (SYN), linezolid (LZD), penicillin (PEN), kanamycin (KAN), erythromycin (ERY), ciprofloxacin (CIP), vancomycin (VAN), lincomycin (LIN), and gentamicin (GEN). The antibiotic concentrations tested are shown in Tables 2, 3.

**Table 2:**
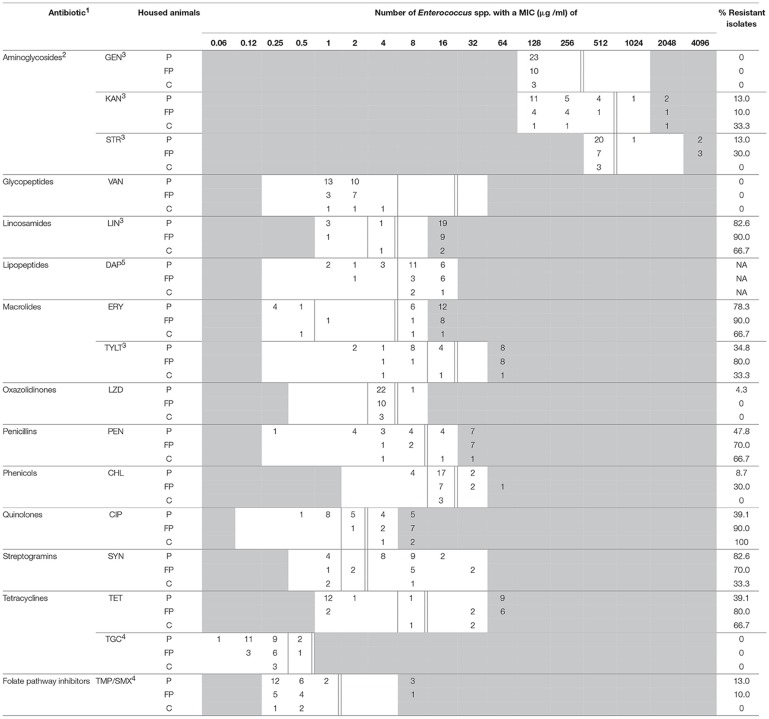
MIC distribution for antimicrobial agents of *Enterococcus* spp. from BAA.

*P, pigs; FP, fattening poultry; C, cattle*.

*^1^Breakpoints are adopted from CLSI (2016) when available; ^2^Breakpoints for all aminoglycosides are high-level resistance; ^3^Breakpoints are established by NARMS (2016); ^4^Breakpoints are based on EUCAST (2016); ^5^Only a susceptible breakpoint is confirmed; the range of trimethoprim/sulfamethoxazole is shown as the trimethoprim concentration. Unshaded areas show the span of the broth microdilution panels. Single vertical bars indicate a susceptibility breakpoint; double vertical bars are breakpoints for resistance*.

*NA, not applicable*.

**Table 3:**
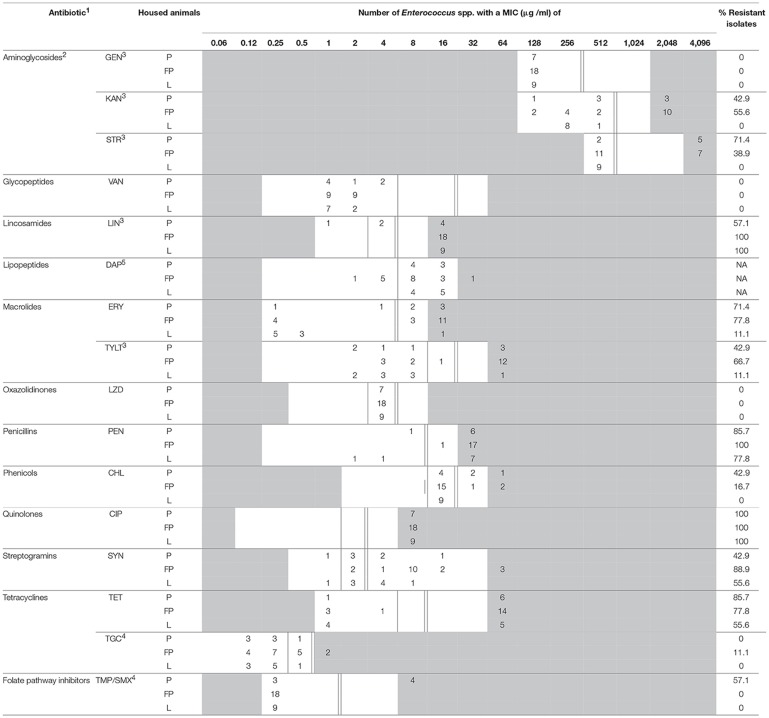
MIC distribution for antimicrobial agents of *Enterococcus* spp. from BAACIP.

P, pigs; FP, fattening poultry; L, laying hens.

*^1^Breakpoints are adopted from CLSI (2016) when available; ^2^Breakpoints for all aminoglycosides are high-level resistance; ^3^Breakpoints are established by NARMS (2016); ^4^Breakpoints are based on EUCAST (2016); ^5^Only a susceptible breakpoint is confirmed; the range of trimethoprim/sulfamethoxazole is shown as the trimethoprim concentration. Unshaded areas show the span of the broth microdilution panels. Single vertical bars indicate a susceptibility breakpoint; double vertical bars are breakpoints for resistance*.

*NA, not applicable*.

Due to the absence of trimethoprim-sulfamethoxazole (TMP/SMX) in the prepared panel, sensitivity to these agents was measured separately. Trimethoprim and sulfamethoxazole (Sigma-Aldrich, co., St. Louis, USA) dissolved in methanol were mixed in sterile broth (ratio 1:19). After dilution, the trimethoprim-sulfamethoxazole suspension was added to blank panels. The concentration ranges are also shown in Tables 2, 3.

Fresh Enterococci broth suspension was prepared, and all panels were incubated at 37°C for 24 hours (CLSI, 2016; EUCAST, 2016). *E. faecium* (DSM 2918) was used as a quality control. The results were read using the VIZION® system (TREK Diagnostik Systems Ltd., West Sussex, UK). According to guidelines of the Clinical and Laboratory Standards Institute (CLSI), tiny buttons of growth were ignored when reading the minimum inhibitory concentration (MIC) of CHL, ERY, LZD, and TET (CLSI, 2016).

Breakpoints were adopted from CLSI (2016) when available. Three aminoglycosides (gentamicin, kanamycin, and streptomycin) were only tested for high-level resistance, and their breakpoints were obtained from the National Antimicrobial Resistance Monitoring System Animal Isolates (NARMS) of the United States Department of Agriculture (NARMS, 2016). The breakpoints for LIN and TYLT were obtained from NARMS as well (NARMS, 2016). The breakpoints for tigecycline and trimethoprim-sulfamethoxazole were obtained from the European Committee on Antimicrobial Susceptibility Testing (EUCAST, 2016). These figures are also included in Tables 2, 3.

### Statistical Analyses

Statistical analyses were performed using SAS 9.4 (SAS Institute Inc., Cary, NC, USA). For each antibiotic, significant differences between the number of resistant isolates from pigs, fattening poultry (broilers, turkeys, ducks), laying hens, and cattle were analyzed by exact conditional logistic regression using the GENMOD procedure. Exact score tests and odds ratios were calculated to estimate significant differences between the animal groups (Stokes et al., 2012). *P*-values ≤ 0.05 were interpreted as statistically significant. Isolates were collected from samples between 1980 and 2015. The CORR procedure was used to test if the number of resistances is associated with the age of isolated *Enterococcus* spp. (Supplementary Table 1). Pearson's and Spearman's correlation coefficients were calculated and considered as significant when *P*-values were ≤ 0.05. Associations between total isolates, isolates from BAA, and isolates from BAACIP were tested.

## Results

### Isolation and Identification of *Enterococcus* spp.

The API® 20 STREP tests identified 70 presumed isolates to *Enterococcus* spp., including 36 from BAA agar and 34 from BAACIP agar. Further identification to species level by MALDI-TOF MS resulted in 64 *E. faecium* isolates, five *E. hirae* isolates and one *E. casseliflavus* isolate. Table 1 shows the origin of isolates and the year of sampling. *E. faecium* was detected in samples from as early as the early 1980s. *E. hirae* was first cultivated from dust from 1989. Enterococci growing on CIP-supplemented media appeared later in 1994. *E. faecium* was detected in dusts from barns occupied with different animal species, whereas *E. hirae* was isolated from only pig barns.

### Frequency of Antimicrobial Resistances in *Enterococcus* spp. Isolates

**Figure 1:**
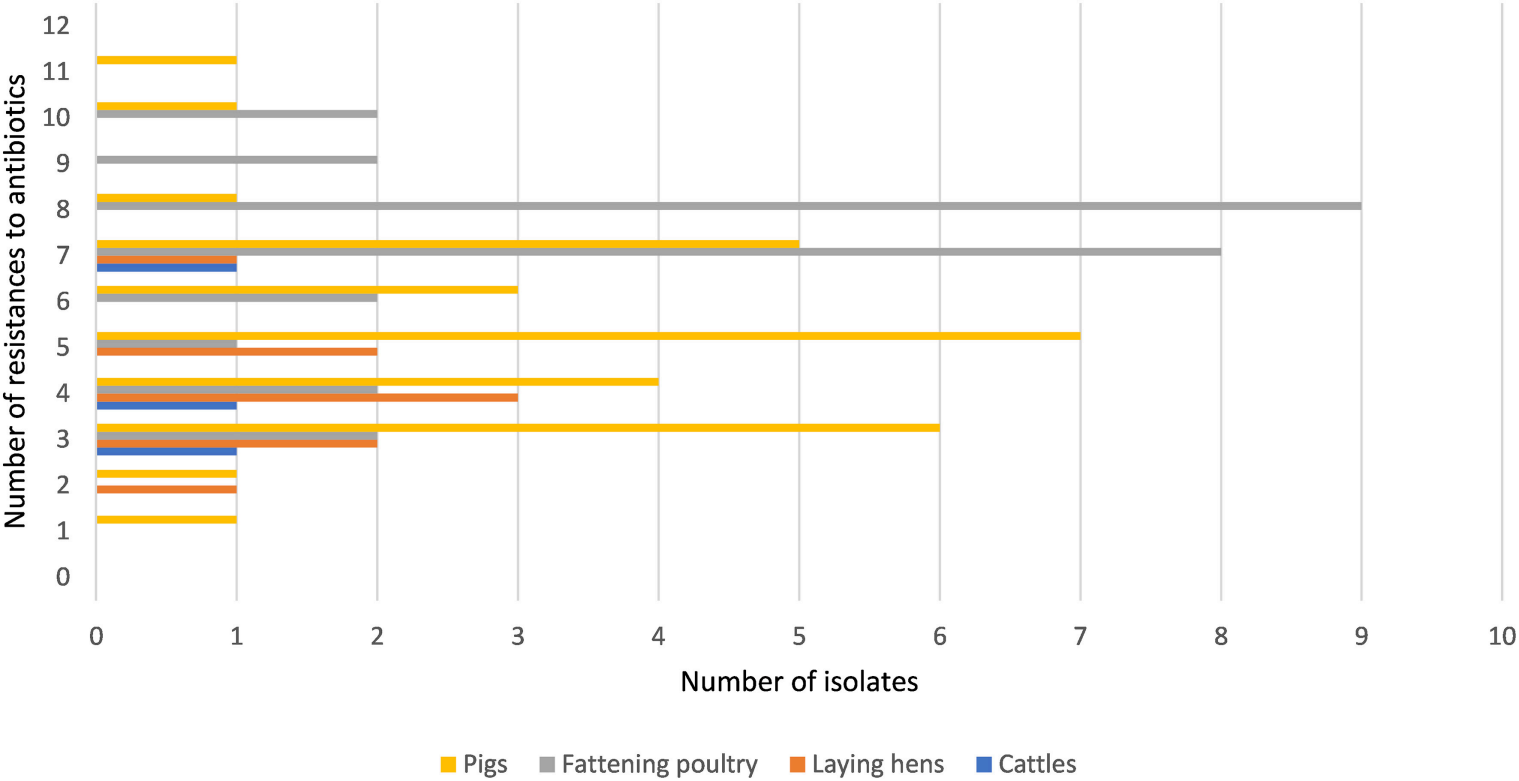
Frequencies of antimicrobial resistances in *Enterococcus* spp. isolates.

Figure 1 shows that all isolates were resistant to one or more of the tested antibiotics. Ninety-six percent (67/70) of all isolates were resistant to three or more antimicrobial agents. Overall, isolates from fattening poultry showed higher numbers of resistances, although a single isolate from a pig barn exhibited the highest number of phenotypic resistances (*n* = 11). Isolates from laying hen houses were resistant to fewer antibiotics compared to isolates from pig and fattening poultry barns. Only three isolates from a cattle barn were included. However, the results in Supplementary Table 1 show that these isolates were resistant to a minimum of three antibiotics from different drug classes.

Seven isolates obtained from dust in fattening poultry barns collected before 2000 were resistant to TYLT. However, for the dust samples collected since 2000, the percentage of resistant isolates was 61.9% (13/21) among fattening poultry. In the isolates from pig farms, the rate of resistant isolates before 2000 was 46.7% (7/15), and then it dropped to 26.7% (4/15) from 2000 onward.

The percentages of SYN-resistant isolates from samples collected before 2000 were 100% (7/7) from fattening poultry barns and 93.3% (14/15) from pig barns. For the younger isolates, the percentages were 76.2% (16/21) and 53.3% (8/15), respectively.

### Correlations Between Age of Isolates and Number of Resistances

Correlation analyses were carried out to investigate associations between the number of phenotypic resistances (total isolates, isolates from BAA, and isolates from BAACIP) and the age of the isolates. No correlations were found for total isolates (Spearman correlation coefficient, ρ = 0.16, *p* = 0.187) and isolates from BAA (ρ = 0.05, *p* = 0.776). A moderate monotonic relationship (ρ = 0.45, *p* = 0.008) was only obtained when the number of resistances and the age of isolates from BAACIP media were compared.

### Antimicrobial Susceptibility

The minimal inhibition concentrations for 16 antimicrobial agents of *Enterococcus* spp. are shown in Table 2 (isolates from BAA) and Table 3 (isolates from BAACIP). All enterococci were sensitive to GEN and VAN. Only a few isolates were not susceptible to LZD and TGC. In contrast, relatively high resistance rates of isolates from both media were calculated for LIN, ERY, PEN, TET, SYN, and CIP. All isolates from BAACIP were resistant to CIP (>4 μg/ml, Table 3), whereas 15 out of 32 isolates from BAA were susceptible to this antibiotic (Table 2). The association between resistant isolates and growing on CIP-containing media was highly significant (Fisher's exact test, *p* < 0.0001). Interestingly, isolates from BAA from the early and late 1980s were already resistant to CIP (Supplementary Table 1).

A breakpoint for daptomycin was not available, but the results indicate that most isolates were not susceptible. Obvious differences appear between the resistance rates of different animal groups in Tables 2, 3. However, the numbers of isolates (pigs = 30, fattening poultry = 28, laying hens = 9, cattle = 3) varied between the groups, which hampered the comparison. Therefore, the significance of differences was calculated by a model, and significant outcomes are summarized in Tables 4, 5.

**Table 4:** Significant differences (*p* ≤ 0.05) of the resistances between isolates from different animal groups.

**Antibiotic**	**CHL**	**CIP**	**ERY**	**KAN**	**LIN**	**PEN**	**SYN**	**STR**	**TET**	**TGC**	**TYLT**
*P*-Value	0.3997	0.0002	0.0003	0.0515	0.0702	0.0297	0.1725	0.1152	0.1676	0.4720	0.0027

*Results of the exact conditional logistic regression*.

**Table 5:** Significant differences in pairwise comparisons of animal groups.

**Animal group**	**Animal group**	**Antibiotic**	**Odds ratio**	**95% confidence limits**	***P*-Value**
Laying hens	Fattening poultry	ERY	32.045	3.235–>999.999	0.0005
Laying hens	Pigs	ERY	23.729	2.518–>999.999	0.0015
Pigs	Fattening poultry	PEN	6.170	1.402–38.856	0.0113
Pigs	Fattening poultry	CIP	22.467	2.914–>999.999	0.0003
Laying hens	Fattening poultry	TYLT	18.290	1.954–930.221	0.0045
Pigs	Fattening poultry	TYLT	4.201	1.262–15.213	0.0161

### Significant Differences of Antibiotic Resistance Rates Between Animal Groups

An exact conditional logistic regression was conducted to analyze the significant differences between the resistances of isolates from different animal groups. As shown in Table 4, the results indicate that the resistances to four antibiotics (ERY, PEN, CIP, and TYLT) were significantly different between isolates from different animal groups. Calculations of the exact odds ratios show significant differences in pairwise comparisons of the animal groups (Table 5). The chances of finding resistant isolates showed a general trend. Dust from fattening poultry barns obviously more often contained isolates resistant to ERY, PEN, CIP, and TYLT. Isolates from pig holdings had a higher chance of being resistant to ERY than those from laying hen houses.

## Discussion

*Enterococcus* spp. were isolated from dust samples using BAA. The preparation of dust samples and the subsequent cultivation had a detection limit of 1,000 cfu/g dust. The method enabled the detection of *Enterococcus* spp. in even the oldest sample. Considering that microbial growth in the samples was not possible under storage conditions (Schulz et al., 2016), this means that the oldest isolate (*E. faecium*) survived 35 years in a stored environmental sample. Analyzing more presumed isolates and using an enrichment method would have probably enhanced the overall detection rate (Ieven et al., 1999). However, isolating *Enterococcus* spp. from all time periods was possible, and we suggest that *Enterococcus* spp. can be suitable indicator bacteria for retrospective studies with contaminated dry material.

The main species of presumed enterococci was *E. faecium*. This species probably belongs to the typical microbiota in feces from farm animals (Hershberger et al., 2005). Furthermore, *E. faecium* tends to survive longer on dry material than other enterococci (Neely and Maley, 2000). Both of these factors may have influenced the predominant isolation of *E. faecium*. Other species detected were *E. hirae* and *E. casseliflavuss*. *Enterococcus hirae* may be part of the intestinal microbiota of pigs (Larsson et al., 2014) and *E. casseliflavus* was detected also in broiler flocks (Stępień-Pyśniak et al., 2016). The survival for more than two decades in dust also indicates a high tenacity of these species.

The number of phenotypic resistances varied between one and 11. Most of the isolates (98.6%) were multidrug resistant (MDR) according to a definition by Frye and Jackson (2013). The number of antibiotic resistances may vary due to the different treatment regimes in animal husbandry. The treatment status of the sampled barns was unknown. For instance, other studies on isolates of MDR *E. faecium* from food animals revealed 31.7% MDR isolates from cattle, 65.8% MDR isolates from broiler chickens, and 84.6% MDR isolates from pigs (EFSA, 2015; Nowakiewicz et al., 2017). Although the studies are not directly comparable, other sets of antibiotics were tested, so the results of our study and the cited studies indicate that MDR *Enterococcus* spp. is probably widespread in farm animal husbandry.

It is noteworthy that the oldest isolate in the present study (isolated in 1981) was resistant to seven different drug classes. A significant association was not found between the age of the isolates and the number of resistances. It is known that bacteria of animal origin can accumulate antimicrobial drug resistances over time (Tadesse et al., 2012). In the present study, younger isolates showed not more resistances than older isolates. In this context, it must be considered very likely that the heterogeneity of the investigated samples, e.g., different numbers of samples from different time periods and different origins, influenced the results. As an example, isolates from laying hens (all sampled in 2005) showed fewer resistances than older isolates from pigs and fattening poultry.

The susceptibility of isolates to different antimicrobial agents varied greatly (Table 2, 3). Enterococci were completely sensitive to VAN and high-level GEN. Only a few isolates were not susceptible to LZD and TGC. There was a relatively high rate of resistance to LIN, ERY, PEN, TET, SYN, and CIP. BAA supplemented with CIP was used to isolate enterococci from dust samples because fluoroquinolone-resistant enterococci were of special interest. Ciprofloxacin was chosen as a representative of fluoroquinolones because it is a common choice for human bacterial diseases and it is closely related to enrofloxacin, which has been was used extensively in animal husbandry (Guardabassi et al., 2008).

Bacteria show cross-resistance to ciprofloxacin and enrofloxacin (Van den Bogaard et al., 2001). Enrofloxacin was first introduced in German animal husbandry in 1989 (Guardabassi et al., 2008). Thus, the results indicate that the occurrence of CIP-resistant enterococci in the early 1980s was not influenced by the treatment of animals. Resistance to fluoroquinolones in bacteria is multifactorial (Redgrave et al., 2014), and the reason for this early occurrence remains unknown. Isolates from supplemented media were significantly more resistant to ciprofloxacin. However, the resistance among 58% of the isolates from non-selective media and the detection in pig and poultry barns and a cattle barn (Table 2) indicate a spread of ciprofloxacin resistance in the farm animal facilities investigated.

High-level resistance breakpoints were used for aminoglycosides because enterococci can prevent aminoglycosides from penetrating the bacterial cell membrane and thus have low-level intrinsic resistance (Zimmermann et al., 1971; EUCAST, 2016). Although high-level resistance against gentamicin was not found, nearly one-third of isolates had high-level resistance to kanamycin and streptomycin. Resistances to these antibiotics in farm animals might result from the wide and long-term usage of aminoglycosides in Europe (EMA, 2014).

Due to serious nosocomial infections, VRE invariably cause concern among researchers. VRE have been isolated in Germany as early as 1987 (Lütticken and Kunstmann, 1988). Vancomycin resistant enterococci have been isolated from food animals in Sweden, the Netherlands, and Germany (Stobberingh et al., 1999; Nilsson et al., 2009; Sting et al., 2013). However, all enterococci isolated in this study were sensitive to vancomycin.

There was a high percentage of LIN-resistant enterococci, especially in isolates from poultry farm dust. Thirty-six isolates (97.3%) of enterococci from dust in broiler, layer, and turkey houses were resistant to LIN. These findings are consistent with those from another study (Maasjost et al., 2015). Lincosamides and macrolides are important therapeutic agents for the treatment of infections in farm animals (Pyörälä et al., 2014). The resistance to ERY was notable in this study. Except for the isolates from laying hen barns, the percentages of ERY-resistant enterococci were all over 60% (Tables 2, 3). Isolates from pig barns and fattening poultry barns had a higher chance of being resistant to ERY than those from laying hen barns (Table 5). It can be assumed that laying hens are generally treated less because of the problem with residues in eggs.

There was a lower percentage of resistance to TYLT, another macrolide, than to ERY (Tables 2, 3). Furthermore, the percentage of TYLT resistant isolates from poultry and pig barns decreased since 2000. It is uncertain whether this observed decrease was influenced by the ban of TYLT as a growth promoter at the end of 1998 in the European Unions (Wegener et al., 1999), but the results show that resistance was still present in isolates from 2015.

Quinupristin/dalfopristin was the first antibiotic for human VRE infections with good clinical effect (Wegener et al., 1999). Virginiamycin and SYN are streptogramins. Due to the “Precautionary Principle,” virginiamycin was prohibited as an antibiotic growth promoter at the same time as TYLT (Casewell et al., 2003). A decrease in resistant isolates has been observed for SYN. However, a more comprehensive study would be necessary to confirm this downtrend.

Over 70% of *Enterococcus* spp. were resistant to PEN. Resistance rates of the same magnitude were detected in *E. faecium* isolates from poultry production environments in the United States (Hayes et al., 2004). These high resistance rates may be due to an induced, intrinsic, low-level resistance of *E. faecium* to PEN (Maasjost et al., 2015). A correlation between penicillin and ciprofloxacin resistance has also been observed (Adela et al., 2004).

Although the rate of CHL resistance was < 20%, it was obviously higher than in other studies in Germany (Peters et al., 2003; Maasjost et al., 2015). Chloramphenicol was forbidden for use in farm animals in Europe in 1994 (Maasjost et al., 2015). However, in the present study resistant isolates occurred sporadically in poultry and pig barns after the ban.

Linezolid has been allowed for clinical use in humans in Europe since 2001 (Seedat et al., 2006). Although LZD can be used in pets, it should be prescribed only in rare cases (Wijesekara et al., 2017). The first LZD-resistant VRE was found in Germany in 2004 (Halle et al., 2004). In our study, no LZD-resistant *E. faecium* was detected. Only one isolate of *E. hirae* was resistance to LZD. Almost all MIC values for LZD were in the intermediate range (Tables 2, 3). Resistance to TMP/SMX was also scarce. In general, resistance to TMP/SMX seems to be rare in Gram-positive bacteria isolated from German farm animals (Schwarz et al., 2013).

All enterococci in this study were resistant to one or more antimicrobials (Figure 1). Approximately 75.0% of isolates from dust from fattening poultry farms were resistant to seven or more antimicrobials compared with only 26.7% from pigs. The resistances to ERY, PEN, CIP, and TYLT were significantly different between isolates from different animal groups (Table 4). In a second step, a statistical model revealed that *Enterococcus* spp. isolated from fattening poultry barns were more often resistant to these antibiotics compared to other animal groups (Table 5). Furthermore, isolates from fattening poultry barns showed the highest rate resistance to multiple antibiotics (Figure 1). These results may be related to the different antibiotic regimes in the environments investigated and suggest that more antibiotics were used in poultry barns.

Metagenomic analyses of environmental samples revealed that antibiotic resistance is an ancient, naturally occurring phenomenon (D'Costa et al., 2011). Although such studies can confirm that genes homologous to resistance genes existed in ancient bacteria, DNA fragments cannot confirm functional resistance against antibiotics (Perron et al., 2015). A study from Perron et al. (2015) revealed functional antibiotic resistance in at last 5,000 years old permafrost. However, whether bacteria survived such a long time or were part of subpopulations remained unknown. This study showed that the long-term survival of enterococci in dust enabled a retrospective view of the phenotypic antimicrobial resistances in isolates from different barns of intensive livestock farming. In comparison to a study from Schulz et al. (2016), the present study detected fluoroquinolone resistant bacteria before these antibiotics were used in farms. The resistance in the absence of fluoroquinolone pressure is likely to be related to the biology of resistance (Redgrave et al., 2014). However, this demonstrates that farm animals can be a reservoir of fluoroquinolone resistant bacteria, although animals came never into contact with these antibiotics. Moreover, it was forbidden to treat laying hens with fluoroquinolones in the European Union (Anonymous, 2002) but all isolates from laying hens in 2005 were resistant to CIP. An eradication of CIP resistant enterococci will not be as simple as prohibiting the use of these agents.

Farmers, animals, and the environment are exposed to dust-bound MDR enterococci shed by carrying animals. Intervention methods such as thoroughly cleaning of all contaminated surfaces in barns are necessary to avoid transmissions. Whether animal strains can be transmitted to humans remains controversial (Donabedian et al., 2006). However, in terms of prevention, farmers might protect themselves by hygiene measures such as changing clothes, appropriate hand hygiene, and wearing dust masks.

## Author Contributions

ML isolated and identified the isolates, conducted the antimicrobial susceptibility testing, and carried out the data analysis. NV programmed the statistical model and analyzed the results. JS planned the study and did the correlation analysis. ML, NK, and JS wrote the manuscript. All authors read and approved the final manuscript.

### Conflict of Interest Statement

The authors declare that the research was conducted in the absence of any commercial or financial relationships that could be construed as a potential conflict of interest.

## Supplementary Material

Supplementary Table 1 Origin, identification, and MIC values of isolates.

## References

[B1] Adela G. Balsalobre L. Ardanuy C. Fenoll A. Pérez-Trallero E. Liñares J. (2004). Fluoroquinolone resistance in penicillin-resistant *Streptococcus pneumoniae* clones, Spain. Emerg. Infect. Dis. 10, 1751–1759. 10.3201/eid1010.040382 15504260 PMC3323274

[B2] Anonymous (2002). COMMISSION REGULATION (EC) No 1181/2002 of 1 July 2002. Official Journal of the European Communities L 172/13.

[B3] Byappanahalli M. N. Nevers M. B. Korajkic A. Staley Z. R. Harwood V. J. (2012). Enterococci in the environment. Microbiol. Mol. Biol. Rev. 76, 685–706. 10.1128/MMBR.00023-12 23204362 PMC3510518

[B4] Casewell M. Friis C. Marco E. McMullin P. Phillips I. (2003). The European ban on growth-promoting antibiotics and emerging consequences for human and animal health. J. Antimicrob. Chemother. 52, 159–161. 10.1093/jac/dkg313 12837737

[B5] CLSI (2016). Performance Standards for Antimicrobial Susceptibility Testing, 26th Edn. CLSI supplement M100S. Wayne, PA: Clinical and Laboratory Standards Institute.

[B6] D'Costa V. M. King C. E. Kalan L. Morar M. Sung W. W. L. Schwarz C. (2011). Antibiotic resistance is ancient. Nature 407, 457–461. 10.1038/nature10388 21881561

[B7] Devrise L. A. Vancanneyt M. Descheemaeker P. Baele M. Van Landuyt H. W. Gordts B. (2002). Defferentiation and identification of *Enterococcus durans, E. hirae and E. villorum*. J. Appl. Microbiol. 92, 821–827. 10.1046/j.1365-2672.2002.01586.x 11972684

[B8] Donabedian S. M. Perri M. B. Vager D. Hershberger E. Malani P. Simjee S.. (2006). Quinupristin-dalfopristin resistance in *Enterococcus faecium* isolates from humans, farm animals, and grocery store meat in the United States. J. Clin. Microbiol. 44, 3361–3365. 10.1128/JCM.02412-05 16954273 PMC1594738

[B9] EFSA (2015). EU Summary Report on antimicrobial resistance in zoonotic and indicator bacteria from humans, animals and food in 2013. EFSA J. 13:4036 10.2903/j.efsa.2013.3196

[B10] EMA (2014). Available online at: http://www.ema.europa.eu/docs/en_GB/document_library/Scientific_guideline/2014/07/WC500170029.pdf (Accessed at July 24, 2017).

[B11] EUCAST (2016). The European Committee on Antimicrobial Susceptibility Testing. Breakpoint Tables for Interpretation of MICs and Zone Diameters. Version 6.0, 2016. Available online at: http://www.eucast.org

[B12] Frye J. G. Jackson C. R. (2013). Genetic mechanisms of antimicrobial resistance identified in *Salmonella enterica, Escherichia coli*, and *Enteroccocus* spp. Isolated from US food animals. Front. Microbiol. 4:135. 10.3389/fmicb.2013.00135 23734150 PMC3661942

[B13] Guardabassi L. Jensen L. B. Kruse H. (eds.). (2008). Guide to Antimicrobial Use in Animals. London: John Wiley & Sons 15:131.

[B14] Halle E. Padberg J. Rosseau S. Klare I. Werner G. Witte W. (2004). Linezolid-resistant *Enterococcus faecium* and *Enterococcus fecalis* isolated from a septic patient: report of first isolates in Germany. Infection 32, 182–183. 10.1007/s15010-004-3009-0 15188081

[B15] Hammerum A. M. (2012). Enterococci of animal origin and their significance for public health. Clin. Microbiol. Infect. 18, 619–625. 10.1111/j.1469-0691.2012.03829.x 22487203

[B16] Hayes J. R. English L. L. Carr L. E. Wagner D. D. Joseph S. W. (2004). Multiple-antibiotic resistance of *Enterococcus* spp. Isolated from commercial poultry production environments. Appl. Environ. Microbiol. 70, 6005–11. 10.1128/AEM.70.10.6005-6011.2004 15466544 PMC522102

[B17] Hershberger E. Oprea S. F. Donabedian S. M. Perri M. Bozigar P. Bartlett P.. (2005). Epidemiology of antimicrobial resistance in enterococci of animal origin. J. Antimicrob. Chemother. 55, 127–130. 10.1093/jac/dkh508 15574473

[B18] Ieven M. Vercauteren E. Descheemaeker P. Van Laer F. Goossens H. (1999). Comparison of direct plating and broth enrichment culture for the detection of intestinal colonization by glycopeptide-resistant enterococci among hospitalized patients. J. Clin. Microbiol. 37, 1436–1440. 10203501 10.1128/jcm.37.5.1436-1440.1999 PMC84795

[B19] Kataoka Y. Umino Y. Ochi H. Harada K. Sawada T. (2014). Antimicrobial susceptibility of enterococcal species isolated from antibiotic-treated dogs and cats. J. Vet. Med. Sci. 76, 1399–1402. 10.1292/jvms.13-0576 24976587 PMC4221175

[B20] Larsson J. Lindberg R. Aspán A. Grandon R. Westergren E. Jacobson M. (2014). Neonatal piglet diarrhoea associated with enteroadherent *Enterococcus hirae*. J. Compar. Pathol. 151, 137–147. 10.1016/j.jcpa.2014.04.003 24915885

[B21] Lebreton F. Willems R. J. L. Gilmore M. S. (2014). Enterococcus diversity, origins in nature, and gut colonization, in Enterococci: From Commensals to Leading Causes of Drug Resistant Infection, eds Gilmore M. S. Clewell D. B. Ike Y. Shankar N (Boston, MA: Massachusetts Eye and Ear Infirmary). Available online at: https://www.ncbi.nlm.nih.gov/books/NBK190427/24649513

[B22] Liu M. (2017). Dust from Livestock Buildings as Reservoirs for Long-Term Survival of Bacteria. [thesis]. University of Veterinary Medicine Hannover. Available online at: http://elib.tiho-hannover.de/dissertations/lium_ws17.pdf

[B23] Lu H. Z. Weng X. H. Li H. Yin Y. K. Pang M. Y. Tang Y. W. (2002). Enterococcus faecium-related outbreak with molecular evidence of transmission from pigs to humans. J. Clin. Microbiol. 40, 913–917. 10.1128/JCM.40.3.913-917.2002 11880415 PMC120277

[B24] Lukasova J. Sustackova A. (2003). Enterococci and antibiotic resistance. Acta Vet. Brno 72, 315–323. 10.2754/avb200372020315

[B25] Lütticken R. Kunstmann G. (1988). Vancomycin-resistant Streptococcaceae from clinical material. Zentralblatt Bakteriol. Mikrobiol. HygieneSeries A Med. Microbiol. Infect. Dis. Virol. Parasitol. 267, 379–382. 10.1016/S0176-6724(88)80054-3 3376618

[B26] Maasjost J. Mühldorfer K. de Jäckel S. C. Hafez H. M. (2015). Antimicrobial susceptibility patterns of *Enterococcus fecalis* and *Enterococcus faecium* isolated from poultry flocks in Germany. Avian Dis. 59, 143–148. 10.1637/10928-090314-RegR 26292548

[B27] Milton A. A. P. Priya G. B. Aravind M. Parthasarathy S. Saminathan M. Jeeva K. (2015). Nosocomial infections and their surveillance in veterinary hospitals. Adv. Anim. Vet. Sci. 3, 1–24. 10.14737/journal.aavs/2015/3.2s,.1.24

[B28] NARMS: National antimicrobial resistance monitoring system animal isolates, (2016). Breakpoints Used for Susceptibility Testing of Enterococcus. Ars.usda.gov. Available online at: https://www.ars.usda.gov/southeast-area/athens-ga/us-national-poultry-research-center/bacterial-epidemiology-antimicrobial-resistance-research/docs/narms-national-antimicrobial-resistance-monitoring-system-animal-isolates/page-3. (Assessed January 18, 2017).

[B29] Neely A. N. Maley M. P. (2000). Survival of enterococci and staphylococci on hospital fabrics and plastic. J. Clin. Microbiol. 38, 724–726. Available online at: https://jcm.asm.org/content/38/2/724.short 10655374 10.1128/jcm.38.2.724-726.2000 PMC86187

[B30] Nilsson O. Greko C. Bengtsson B. (2009). Environmental contamination by vancomycin resistant enterococci (VRE) in Swedish broiler production. Acta Vet. Scand. 51:49 10.1186/1751-0147-51-49 PMC2794861 19954525

[B31] Nowakiewicz A. Ziółkowska G. Zięba P. Gnat S. Trościańczyk A. Adaszek Ł. (2017). Characterization of multidrug resistant E. fecalis strains from pigs of local origin by ADSRRS-fingerprinting and MALDI-TOF MS; Evaluation of the compatibility of methods employed for multidrug resistance analysis. PloS ONE 12:e0171160 10.1371/journal.pone.0171160 28135327 PMC5279778

[B32] Perron G. P. Whyte L. Turnbaugh P. J. Goordial J. Hanage W. P. Dantas G.. (2015). Isolated from ancient arctic soil exposes diverse resistance mechanisms to modern antibiotics. PloS ONE 10:e0069533. 10.1371/journal.pone.0069533 25807523 PMC4373940

[B33] Peters J. Mac K. Wichmann-Schauer H. Klein G. Ellerbroek L. (2003). Species distribution and antibiotic resistance patterns of enterococci isolated from food of animal origin in Germany. Int. J. Food Microbiol. 88, 311–314. 10.1016/S0168-1605(03)00193-4 14597003

[B34] Public Health England (2014). Identification of Streptococcus species, Enterococcus species and Morphologically Similar Organisms. GOV.UK. Available online at: https://www.gov.uk/government/uploads/system/uploads/attachment_data/file/369824/ID_4i3.pdf (Accessed March 1, 2017).

[B35] Pyörälä S. Baptiste K. E. Catry B. Van Duijkeren E. Greko C. Moreno M. A.. (2014). Macrolides and lincosamides in cattle and pigs: use and development of antimicrobial resistance. Vet. J. 200, 230–239. 10.1016/j.tvjl.2014.02.028 24685099

[B36] Redgrave L. S. Sutton S. B. Webber M. A. Piddock L. J. (2014). Fluoroquinolone resistance: mechanisms, impact on bacteria, and role in evolutionary success. Trends Microbiol. 22, 438–445. 10.1016/j.tim.2014.04.007 24842194

[B37] Schulz J. Ruddat I. Hartung J. Hamscher G. Kemper N. Ewers C. (2016). Antimicrobial-resistant escherichia coli survived in dust samples for more than 20 Years. Front. Microbiol. 7:866. 10.3389/fmicb.2016.00866 27375587 PMC4901058

[B38] Schwarz S. Kadlec K. Silley P. (2013). Antimicrobial Resistance in Bacteria of Animal Origin. Steinen: ZETT-Verlag.

[B39] Seedat J. Zick G. Klare I. Konstabel C. Weiler N. Sahly H. (2006). Rapid emergence of resistance to linezolid during linezolid therapy of an Enterococcus faecium infection. Antimicrob. Agents Chemother. 50, 4217–4219. 10.1128/AAC.00518-06 16982791 PMC1694002

[B40] Stepień-Pyśniak D. Hauschild T. Różański P. Marek A. (2017). MALDI-TOF mass spectrometry as a useful tool for identification of *Enterococcus* spp. from wild birds and differentiation of closely related species. J. Microbiol. Biotechnol. 27, 1128–1137. 28285496 10.4014/jmb.1612.12036

[B41] Stępień-Pyśniak D. Marek A. Banach T. Adaszek L. Pyzik E. Wilczyński J.. (2016). Prevalence and antibiotic resistance of Enterococcus strains isolated from poultry. Acta Vet. Hungar. 64,148–163. 10.1556/004.2016.016 27342087

[B42] Sting R. Richter A. Popp C. Hafez H. M. (2013). Occurrence of vancomycin-resistant enterococci in turkey flocks. Poultry Sci. 92, 346–351. 10.3382/ps.2012-02652 23300299

[B43] Stobberingh E. van den Bogaard A. London N. Driessen C. Top J. Willems R. (1999). Enterococci with glycopeptide resistance in turkeys, turkey farmers, turkey slaughterers, and (sub) urban residents in the south of The Netherlands: evidence for transmission of vancomycin resistance from animals to humans? Antimicrob. Agents Chemother. 43, 2215–2221. 10.1128/AAC.43.9.2215 10471567 PMC89449

[B44] Stokes M. E. Davis C. S. Koch G. G. (2012). Categorical Data Analysis Using SAS (Cary, NC: SAS Institute).

[B45] Tadesse D. A. Zhao S. Tong E. Ayers S. Singh A. Bartholomew M. J. (2012). Antimicrobial drug resistance in *Escherichia coli* from humans and food animals, United States, 1950–2002. Emerg. Infect. Dis. 18:741 10.3201/eid1805.111153 PMC3358085 22515968

[B46] Thermofisher.com (2017). Product Specification Sheet/Enterococcus Selective Agar (BAA). Available online at: https://tools.thermofisher.com/content/sfs/brochures/PO5062A.pdf (Accessed July 5, 2017).

[B47] Van den Bogaard A. E. London N. Driessen C. A. G. G. Stobberingh E. E. (2001). Antibiotic resistance of fecal Escherichia coli in poultry, poultry farmers and poultry slaughterers. J. Antimicrob. Chemother. 47, 763–771. 10.1093/jac/47.6.763 11389108

[B48] Wegener H. C. Aarestrup F. M. Jensen L. B. Hammerum A. M. Bager F. (1999). Use of antimicrobial growth promoters in food animals and Enterococcus faecium resistance to therapeutic antimicrobial drugs in Europe. Emerg. Infect. Dis. 5:329. 10.3201/eid0503.990303 10341169 PMC2640785

[B49] Wiedemann B. Heisig P. (1999). Bakterielle resistenz gegenüber chinolonen (*Ciprofloxacin*). Chemother. J. 8, 99–107.

[B50] Wijesekara P. N. K. Kumbukgolla W. W. Jayaweera J. A. A. S. Rawat D. (2017). Review on usage of vancomycin in livestock and humans: maintaining its efficacy, prevention of resistance and alternative therapy. Vet. Sci. 4:6. 10.3390/vetsci4010006 29056665 PMC5606620

[B51] Zimmermann R. A. Moellering R. C. Weinberg A. N. (1971). Mechanism of resistance to antibiotic synergism in enterococci. J. Bacteriol. 105, 873–879. 4994038 10.1128/jb.105.3.873-879.1971 PMC248512

